# Heavily Calcified Esthesioneuroblastoma in a 72-year-old

**DOI:** 10.7759/cureus.4298

**Published:** 2019-03-22

**Authors:** Robert Maurer, April Henry, Timothy Maurer, Ghazal Staity, Nicole Williams, Neerav Goyal, Brad Zacharia

**Affiliations:** 1 Neurosurgery, Penn State Milton S. Hershey Medical Center, Hershey, USA; 2 Osteopathy, Philadelphia College of Osteopathic Medicine, Philadelphia, USA; 3 Pathology, Penn State Milton S. Hershey Medical Center, Hershey, USA; 4 Otolaryngology, Penn State Milton S. Hershey Medical Center, Hershey, USA

**Keywords:** esthesioneuroblastoma, olfactory neuroblastoma

## Abstract

A 72-year-old female presented with the complaint of declining vision. Workup included magnetic resonance imaging (MRI) which revealed a large enhancing mass extending into the nasal cavity, nasopharynx, and anterior cranial fossa. Computed tomography (CT) imaging revealed extensive calcification. Subsequent endonasal biopsy revealed the tumor to be an unusually calcified esthesioneuroblastoma (ENB). The patient elected for palliative measures. The case provides a basis for a discussion on rare esthesioneuroblastomas and highlights the possibility of extensive calcification on such tumors.

## Introduction

We present the case of a 72-year-old female with a complaint of declining vision. Workup revealed an extensively calcified large sino-nasal mass. Subsequent endonasal biopsy revealed the tumor to be an unusually calcified esthesioneuroblastoma (ENB). An esthesioneuroblastoma, also known as an olfactory neuroblastoma, is a rare intra-nasal tumor with relatively limited literature dedicated to its presentation, pathology, and prognosis [1]. This case highlights an unusual radiographic appearance that has not been described earlier for such a tumor. It also provides a framework for reviewing the current understanding of these rare tumors.

## Case presentation

A 72-year-old woman with a history of hypertension, hyperlipidemia, gastroesophageal reflux disease, gout, and polymyalgia rheumatica, and a family history of cancer began noticing a gradual loss of vision in both eyes over the course of one year. The declining vision was initially attributed to cataracts, and the patient underwent surgical intervention without noticeable improvement. Following the cataract surgery, the patient had persistent and progressive loss of vision, however, a retinal specialist did not identify any retinal pathology. Further investigation of her vision loss revealed coinciding hearing loss, prompting an MRI and subsequent referral to neurosurgery.

Upon evaluation by neurosurgery, she was found to have fully intact facial symmetry, cognitive function, and upper and lower extremity strength and sensation. Apart from the aforementioned vision and hearing issues, the patient also noticed occasional epistaxis and sinus congestion, which had been treated as a sinus infection several times over the previous year. She was also found to have disconjugate gaze along with a significantly proptotic left eye with 20/60 vision. She denied any headaches, personality changes, focal weakness, numbness, or tingling.

**Figure 1:**
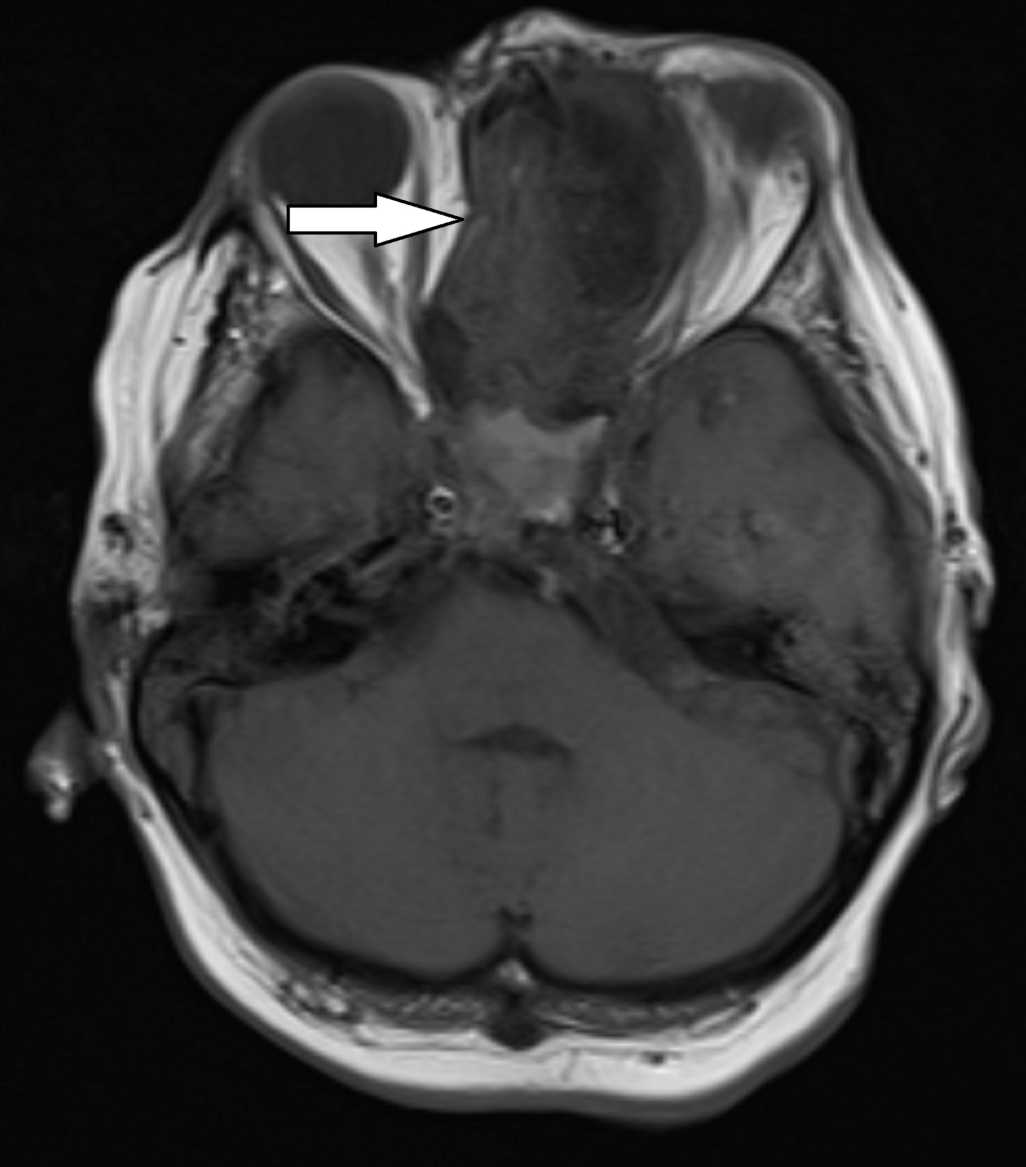
T1 weighted axial MRI

**Figure 2:**
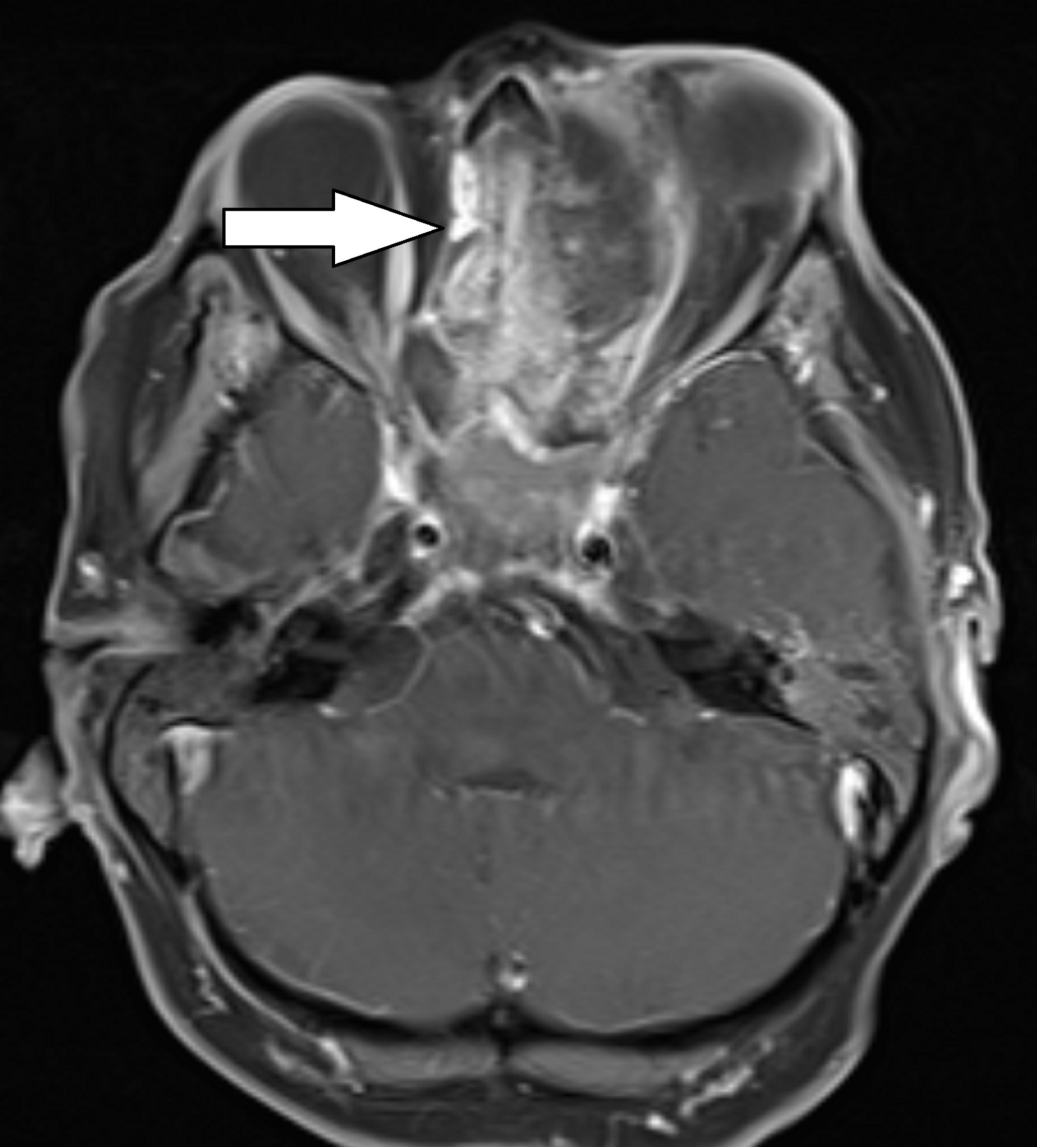
T1 weighted post-contrast axial MRI

**Figure 3:**
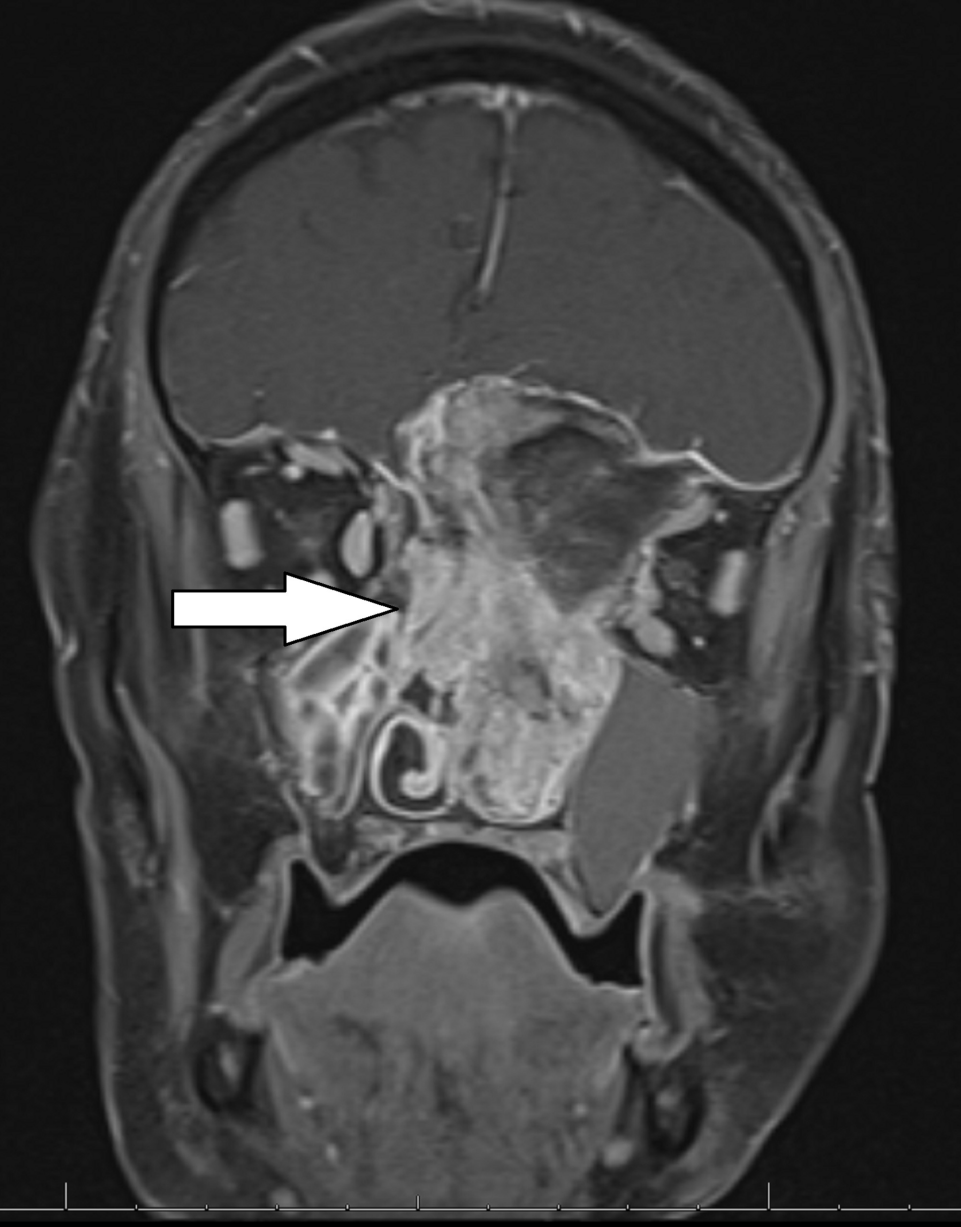
T1 weighted post-contrast coronal MRI

MRI with contrast showed a large enhancing mass, with possible intrinsic bone formation, measuring 7.6 x 2.2 x 6.3 cm (Figures 1-3). The mass extended into the left nasal cavity, inferiorly into the nasopharynx, and superiorly into the anterior cranial fossa. It was noted that there was a destruction of the ethmoid sinus along with mass effects on the left medial rectus muscle and the left optic nerve without an abnormal signal in the optic nerves. The mass displaced the optic chiasm superiorly.

**Figure 4:**
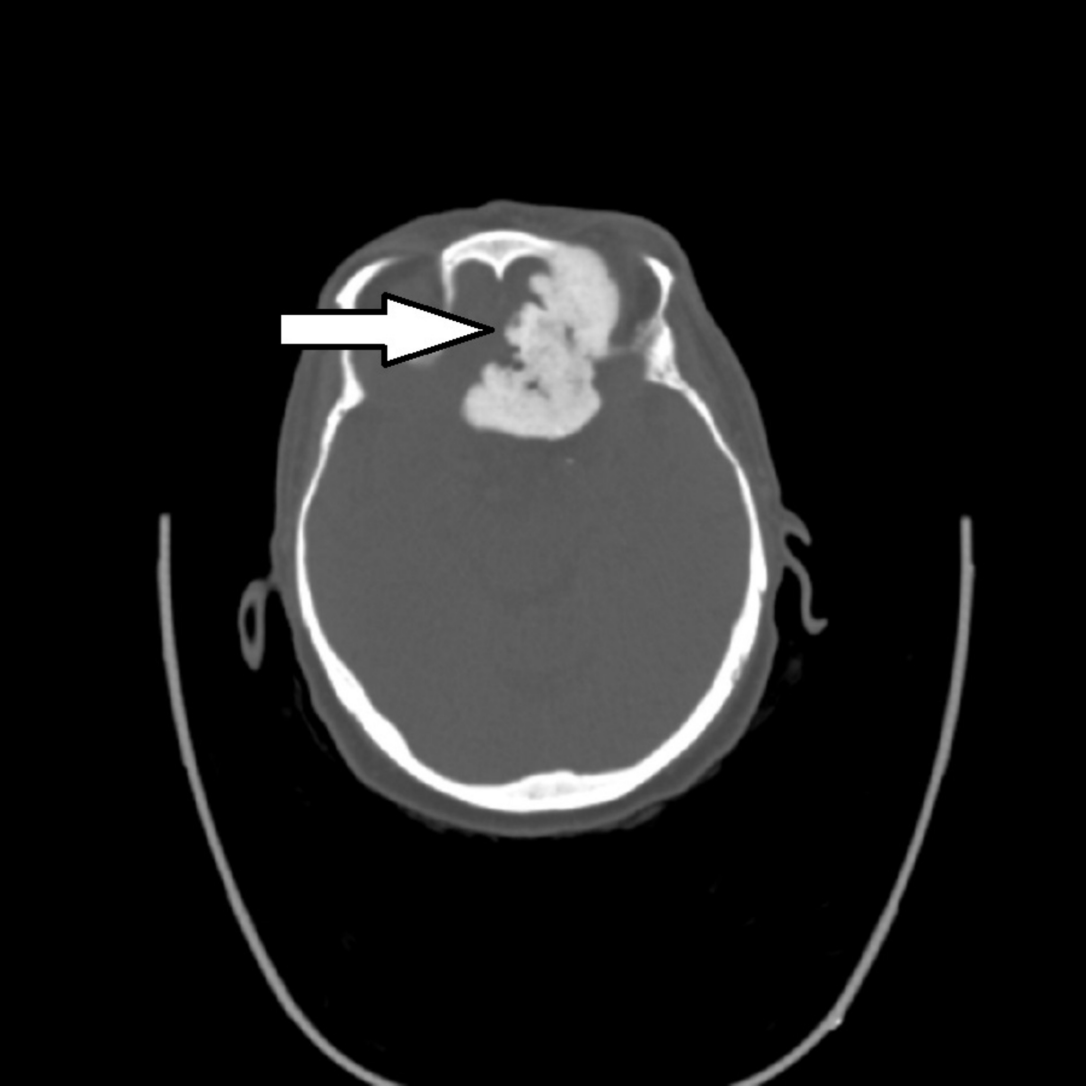
Axial non-contrast CT

**Figure 5:**
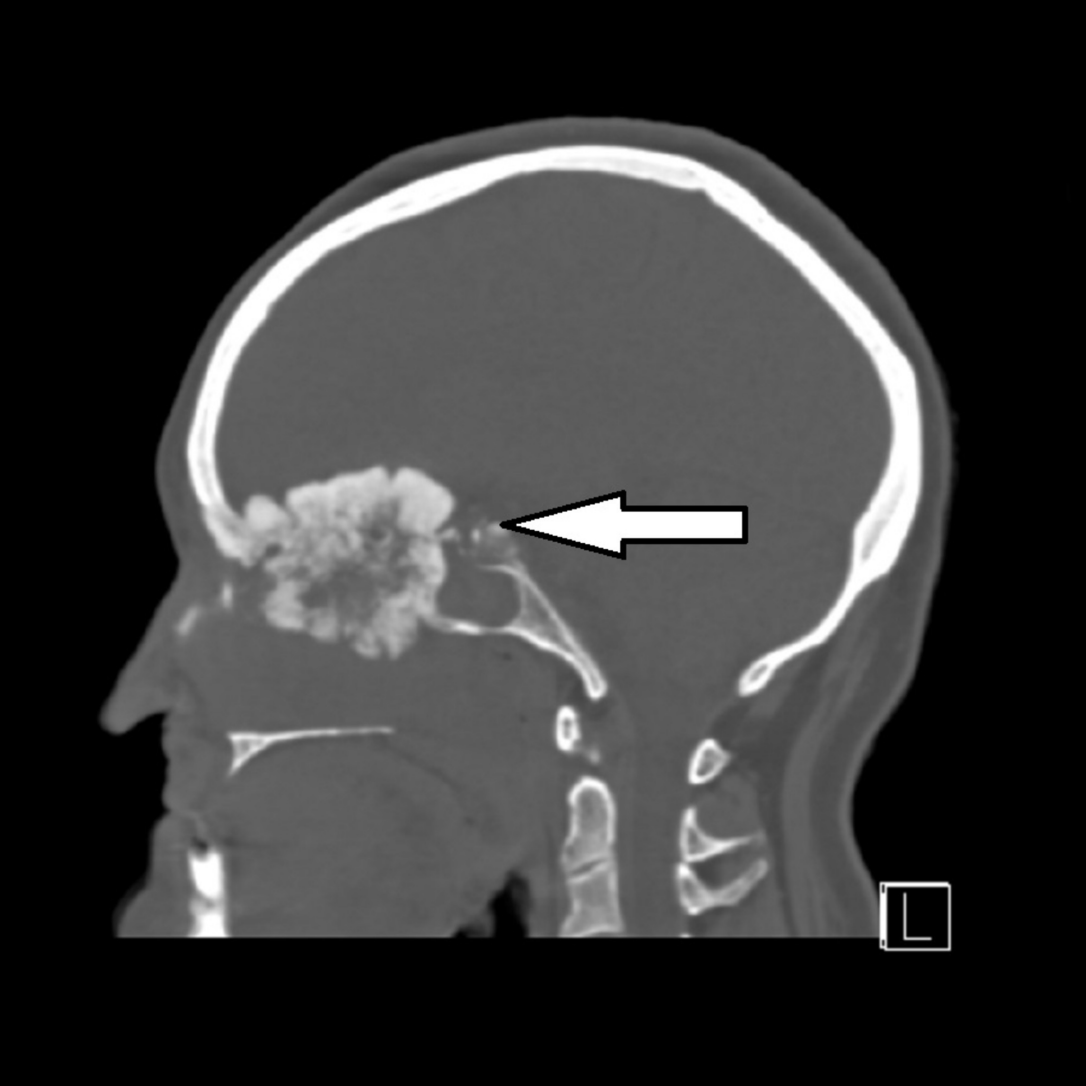
Sagittal non-contrast CT

CT without contrast was performed to further evaluate the tumor and facilitate potential operative planning (Figures 4-5) The results of the CT scan showed a large bone-forming mass centered in the ethmoid bone, invading the ethmoid sinuses, nasal cavity, left frontal lobe, planum sphenoidale, optic canals with intracranial extension, and left orbit, with retained secretions in the sinuses. A developing mucocele was present in the sphenoid sinus associated with thinning of the bony walls. Based on imaging findings, the differential included an undifferentiated sinonasal carcinoma, osteosarcoma, or, less likely, esthesioneuroblastoma.

**Figure 6:**
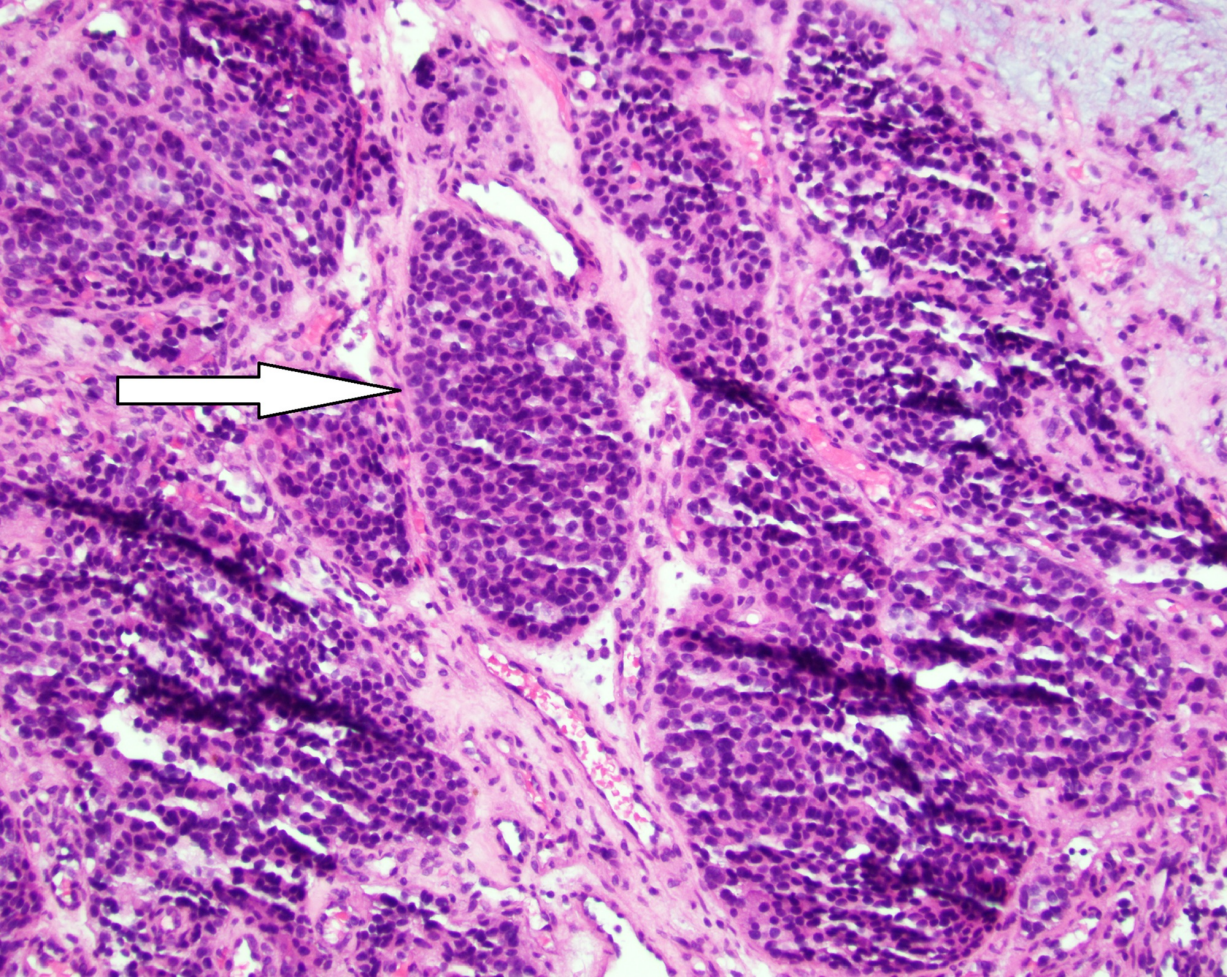
H&E staining of tumor at 200x magnification

**Figure 7:**
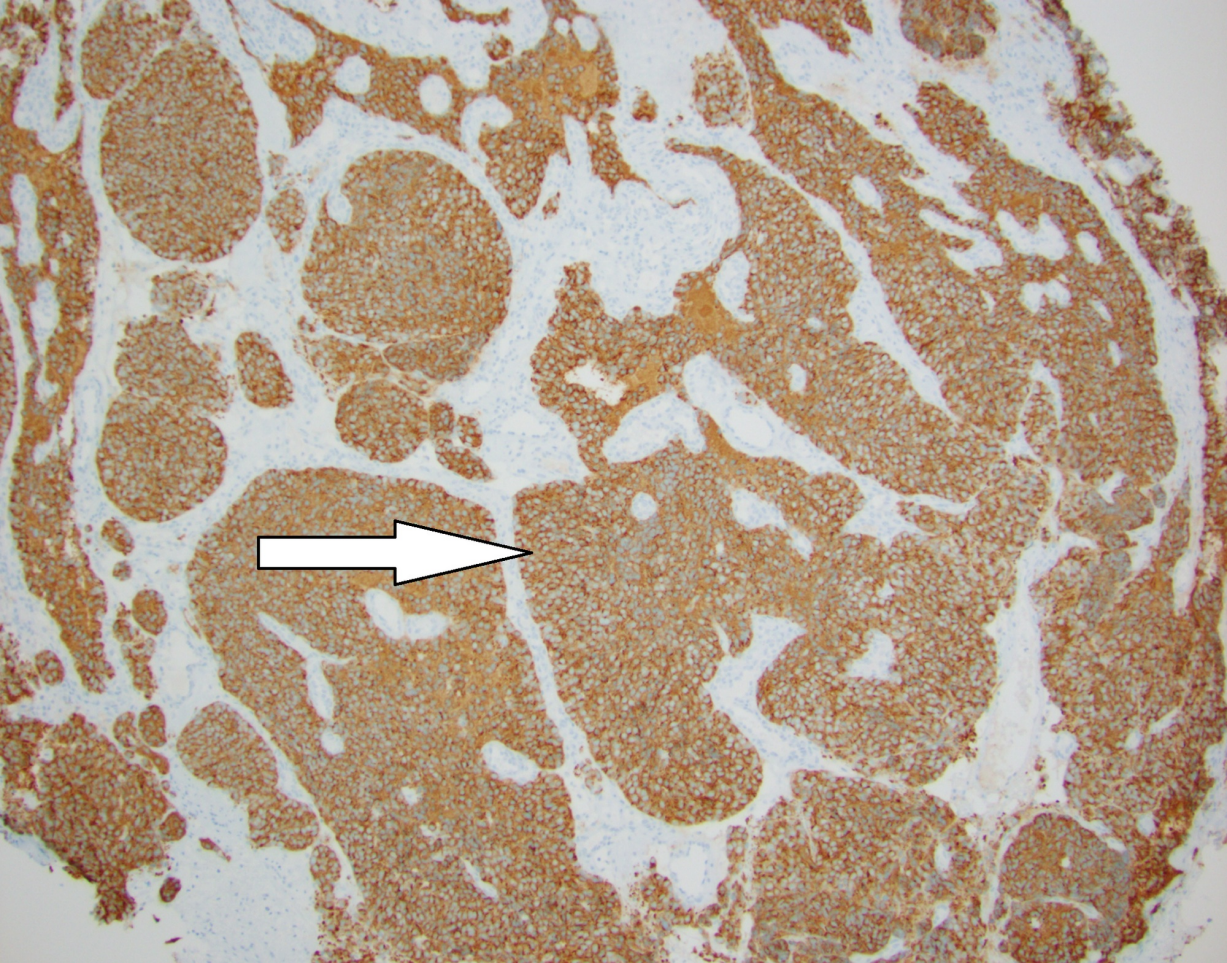
Synaptophysin stain of tumor at 100x magnification

A biopsy of the soft tissue nasal mass was obtained and submitted to the pathology department for evaluation. Hematoxylin and eosin (H&E) stained slides showed sclerotic edematous fibrous tissue infiltrated by lobules of cohesive basophilic tumor cells with a focal pseudorosette architecture. The tumor cells have a high nuclear to cytoplasmic ratio with scant eosinophilic cytoplasm, inconspicuous nucleoli, and scattered mitotic figures (Figure 6). Necrosis was not identified. Immunohistochemical stains demonstrated tumor cells strongly positive for CD56 and synaptophysin with focal chromogranin staining (Figure 7). An S100 stain highlighted peripheral sustentacular cells. Immunohistochemical stains CD99, CD45, and AE1/3 were all negative. In situ hybridization for Epstein Barr virus ribonucleic acid (RNA) was negative. The tumor morphology and immunophenotype confirmed the diagnosis of esthesioneuroblastoma.

After discussing the findings of the radiology studies, biopsy results, and treatment options available for esthesioneuroblastoma, the patient declined treatment of the tumor and was referred to hospice for end-of-life care.

## Discussion

Esthesioneuroblastomas (ENBs), more commonly referred to as olfactory neuroblastomas, were first characterized in 1924 and remain relatively uninvestigated due to the low incidence rates [1]. ENBs approximately account for 3% of all intranasal tumors with an incidence rate of about 0.4 per million in the general population [2-3]. Although the precise origins of ENB tumor growth are unknown, general consensus suggests that it may originate from the basal neural crest cells of the olfactory mucosa [1,4-5]. Previous research suggested that ENB incidences display a bimodal distribution with regards to age [6], however, more recent findings indicate the disease presents across the lifespan with peak incidence occurring in the sixth decade [1,3,7]. Patients afflicted with ENB most commonly present with unilateral symptoms, including epistaxis, nasal obstruction, decreased olfactory function, diplopia, and proptosis, many of which can be associated with benign conditions, making diagnosis difficult [4,8-11].

On radiographic studies, ENB is classically a soft tissue mass. Frequently described as a “dumbbell-shaped” mass filling the ethmoid sinus, with an extension through the cribriform plate into the anterior cranial fossa, ENBs appear as homogenous, contrast-enhancing lesions on both CT and MR imaging. CT imaging can aid in assessing the extent of bony erosion of the cribriform plate and lamina papyracea. Contrast-enhanced MRI studies reveal the extent of the tumor and can be useful for delineating adjacent soft tissue structures [12]. The radiographic appearance presents a broad differential diagnosis which should include carcinomas, sarcomas, melanoma, rhabdomyosarcoma, and malignant lymphoma. Less aggressive entities, which could mimic the appearance of ENBs, include benign cysts and tumors, such as inverting papilloma, hemangioma, and nasal polyps as well as neurogenic tumors, such as neurofibroma, ganglioneuroma, nasal meningocele, and encephalocele [13-14].

A biopsy is required to confirm the diagnosis of ENB. Microscopically, the tumor appears as a “small round blue cell tumor” that can be difficult to differentiate from other tumors, such as sinonasal undifferentiated carcinoma, sinonasal small cell neuroendocrine carcinoma, rhabdomyosarcoma, Ewing sarcoma/primitive neuroectodermal tumor (PNET), lymphoma, and malignant melanoma on tumor morphology alone [15]. Accurate diagnosis requires ancillary testing with immunohistochemical stains demonstrating positive staining for neuroendocrine markers (chromogranin, CD56, synaptophysin, and neuron-specific enolase (NSE)) and S-100 positive sustentacular cells lining the periphery of tumor lobules [16].

Treatment involves maximal safe surgical resection with adjuvant radiotherapy. Chemotherapy is usually reserved as a palliative measure for those tumors deemed inoperable or for recurrent or metastatic lesions [16].

Here, we present a unique case of a highly calcified ENB tumor. To our knowledge, this is the first reported case of such an extensively calcified ENB tumor. This case has significant implications for the diagnosis and management of the disease. The possibility that ENBs can become calcified should be considered for any patient presenting with calcified tumors of the ethmoid sinus and anterior skull base. An appropriately broad differential diagnosis should always be considered to avoid misdiagnoses, which may lead to unnecessary tests or potentially ineffective treatments. This case demonstrates an important radiographic variant of ENB relevant to neurosurgeons, otolaryngologists, and radiologists who may encounter sinonasal tumors. In addition, it raises the question of why some olfactory neuroblastomas remain as soft tissue while others become calcified and highlights the need for further research to identify and treat this rare disease.

## Conclusions

Esthesioneuroblastoma is a rare sinonasal tumor that can present with extensive calcification on imaging studies. An appropriately broad differential diagnosis is required to ensure timely diagnosis and management.
